# Candidate Sites for Millimeter and Submillimeter Ground-Based Telescopes: Atmospheric Rating for the Eurasian Submillimeter Telescopes Project

**DOI:** 10.3390/s25072144

**Published:** 2025-03-28

**Authors:** Artem Y. Shikhovtsev, Pavel G. Kovadlo, Philippe Baron

**Affiliations:** 1 Institute of Solar-Terrestrial Physics, The Siberian Branch of the Russian Academy of Sciences, Irkutsk 664033, Russia; kovadlo2006@rambler.ru; 2 National Institute of Information and Communications Technology (NICT), Koganei 184-8795, Japan; baronph@gmail.com

**Keywords:** millimeter astroclimate, millimeter telescope, atmospheric effects, methods, data analysis, site testing

## Abstract

Modern sensing technologies used in the field of ground-based telescopes still present several challenges. First of all, these challenges are associated with the development of new-generation instruments for astronomical observations and with the influence of Earth’s atmosphere on radiation in various ranges of the electromagnetic spectrum. The atmosphere is often the main factor determining the technical characteristics of the instruments in both the optical and millimeter ranges. In particular, for millimeter/submillimeter telescopes, water vapor is the main gas that determines atmospheric opacity. The correct assessment of water vapor makes it possible to estimate the optical opacity of the atmosphere and, on this basis, the capabilities of the millimeter/submillimeter telescope and the limitations of its use in the mode of very long baseline interferometry. Many studies seek to effectively characterize water vapor content and dynamics for site-testing purposes regarding ground-based millimeter and submillimeter telescope application. In the present article, we study the water vapor content within a fairly large region, which has been poorly covered in the modern literature. Based on the ERA-5 reanalysis data as a site-testing-oriented tool, we obtained spatial distributions of the precipitable water vapor (PWV) within a large region ($20^{\circ }\;N$–$70^{\circ }\;N$, $35^{\circ }\;E$–$120^{\circ }\;E$). These distributions of PWV were corrected with respect to the characteristic vertical scale of water vapor $H_{eff}$ and the relative height difference in the grid nodes in the ERA-5. The calculated values of PWV are highly correlated with the Global Navigation Satellite System-derived PWV, with Pearson coefficients greater than 0.9. Using the refined estimations, we determined the median values of atmospheric opacities corresponding to new prospective sites (Khulugaisha Peak and Tashanta) as well as the Special Astrophysical Observatory (the key astronomical observatory in Russia). Together with Ali in China, Khulugaisha Peak and Tashanta are considered by us as potential sites for the placement of a millimeter/submillimeter telescope within the framework of the project of the Eurasian Submillimeter Telescopes. The results obtained in this paper suggest promising atmospheric conditions for astronomic observations, at least in the millimeter range. In particular, we believe that this study will be a basis for the Eurasian Submillimeter Telescopes (ESMT) project, within the framework of which it is assumed to create a number of ground-based millimeter/submillimeter telescopes.

## 1. Introduction

In the world, there are two particularly important fields of ground-based astronomy. The first field is adaptive optics, which increases the informativeness of astronomical images recorded with ground-based optical and infrared telescopes [1,2,3,4]. World experience shows that the correction of phase turbulent distortions makes it possible to minimize negative atmospheric effects (primarily associated with atmospheric optical turbulence) and approach the ultimate resolution of the telescope. It should be noted that, for infrared telescopes, the key atmospheric quantity, in addition to the characteristics of optical turbulence [5,6], is the content of water vapor within the atmospheric column.

The second area includes a key technology of astronomical observations, which is based on the use of the interferometry principle. By linking together observation data, for example, on spaced radio telescopes, it is possible to achieve significant progress in increasing spatial resolution. In particular, Very Long Baseline (VLBL) demonstrates high scientific potential. For example, for a wavelength of 2 mm and a base of 10,000 km, the potential resolution is about 41 microarcsec; with a decrease in wavelength to 1 mm, the resolution improves to values of about 20 microarcsec.

A certain range of scientific tasks based on VLBL data have been established [7]. One of them is related to increasing the observation potential of the innermost parts of accretion disks and jets around supermassive black holes. Another interesting scientific task is to obtain high-resolution images of bright maser sources, which is important for studying well-known regions of a high-mass star formation containing these bright sources. For millimeter-wave observations, there are also many other compact sources that are potential targets for VLBI applications. These include transient sources such as supernovae, gamma-ray bursts, and microquasars. Such observations should be very interesting since the highest frequencies correspond to the earliest phases of any eruption.

Improvements in our understanding of cosmic phenomena physics are associated with the development of ground-based astronomical infrastructure, the search for new sites on Earth, and the creation of next-generation astronomical instruments. The global community is increasingly involved in the process of organizing complex astronomical observations and creating new measuring instruments [8,9,10,11,12,13]. Studies on water vapor content, air temperature, and wind statistics are important for the selection of new sites that are suitable for millimeter- and submillimeter-wavelength astronomy [14].

For the registration of supermassive black hole images, the Event Horizon Telescope (EHT), a network of antennas on Earth, based on VLBL interferometry, was created [15]. In 2017, the configurations of the network included seven antennas at five sites and eight antennas at six sites across the globe for the observation of M87∗ and Sgr A∗, respectively. Today, with the aim of improving image quality, it is necessary to increase the number of telescopes located at large distances from each other and, thus, to expand the network.

The project “Eurasian Submillimeter Telescopes” proposes the creation of new millimeter/submillimeter telescopes, including in the territory of Russia [16]. The technical characteristics of submillimeter and millimeter ground-based telescopes depend on the atmospheric conditions and, above all, on the water vapor and oxygen content along the line of sight. The estimation of the potential observation time regarding submillimeter and millimeter ground-based telescopes requires preliminary modeling of atmospheric conditions and conducting in situ measurements in selected (or existing) optimal locations for astronomical observations.

In [17], the authors claimed “on Earth’s surface, there are only a handful of high-quality astronomical sites that meet the requirements for very large next-generation facilities”. This expression, in our opinion, is not entirely fair. Indeed, internationally world-class sites are located in such regions as the Atacama Desert in northern Chile, Mauna Kea in the Hawaiian Islands, and La Palma in the Canary Islands [18]. Extremely low values of PWV are recorded at these sites. For example, at the Chile plateau, January and February are the wetter months of the year, while June through August, the driest months, offer the best conditions for submillimeter astronomy observations [19]. The medians of PWV are 2.4 mm and 2.1 mm in January and February, respectively. During the driest months, PWV ≤ 1 mm. The results of other studies show that there are additional locations on Earth that are suitable for installing new telescopes. For example, we can note Sanglokh, Maidanak, Suffa, Muztagh-Ata, and Ali [20,21]. Water vapor content statistics for these sites will be given below.

In this paper, we focus on two new sites: Khulugaisha Peak ($51^{\circ }44^{\prime }55^{\prime \prime }$ N$,\;100^{\circ }59^{\prime }7^{\prime \prime }$ E) and Tashanta ($49^{\circ }43^{\prime }02^{\prime \prime }$ N$,\;89^{\circ }11^{\prime }27^{\prime \prime }$ E). In 2022, we showed that Khulugaisha Peak and Tashanta are two new promising sites that are suitable for the millimeter telescopes located in the Sayan Mountains and Altai, respectively [22]. Direct measurements of atmospheric characteristics confirm our data [23].

For comparative purposes, the location of the Big Telescope Alt-Azimuthal (BTA) of the Special Astrophysical Observatory (SAO) is also considered. The SAO is one of the largest astronomical centers in Russia and houses the RATAN-600 radio telescope as well as the Big Telescope Alt-Azimuthal, which features a 605 cm primary mirror. The SAO is located near Mount Pastukhova in the northern part of the Caucasus Mountains at a height of 2070 m above sea level. The geographic coordinates of the BTA are $43^{\circ }39^{\prime }12^{\prime \prime }$ N, $41^{\circ }26^{\prime }30^{\prime \prime }$ E. We should emphasize that the prospects for developing the observational base at the SAO for astrophysical research in the millimeter-wave range are discussed in [24,25].

## 2. Data Used

Water vapor is the main gas that leads to the absorption of millimeter and submillimeter radiation in the atmosphere. The study of its spatiotemporal variability, as well as the assessment of statistical characteristics of the water vapor content in the atmospheric column and at different heights, are important tasks of millimeter and submillimeter astronomy. In solving these problems, reanalysis databases are becoming increasingly popular and represent one of the possible alternatives to measurements due to the globality, easy accessibility, a certain universality, and duration of the data [26].

For estimation of precipitable water vapor (PWV), we used a global set of publicly available periodically updated ERA-5 reanalysis data covering a time period from 1940 to the present. The temporal resolution of these data is 1 h; the spatial resolution is 0.$25^{\circ }$. The data are provided by the European Center for Medium-Range Weather Forecast Reanalysis [27]. When estimating PWV using ERA-5 data, errors mainly originate from the effects of limited spatial resolution and, perhaps, due to the complex physics of the formation of air humidity fields in mountainous areas where telescopes are usually located. At least the results of mesoscale modeling indicate a high degree of locality in the formation of meteorological fields in these areas. Due to the low spatial resolution, reanalysis does not reproduce forced convection, mesoscale vortex formations, and poorly describes the stratification of the low atmospheric layer, local air transfer, and the dynamics of the atmospheric boundary layer [28,29,30]. This is especially noticeable in mountainous regions and for areas with a sparse network of surface meteorological stations and radiosonde stations [28]. Also, errors in the ERA-5 data depend on the season, large-scale atmospheric processes, and other factors [31].

**Figure 1:**
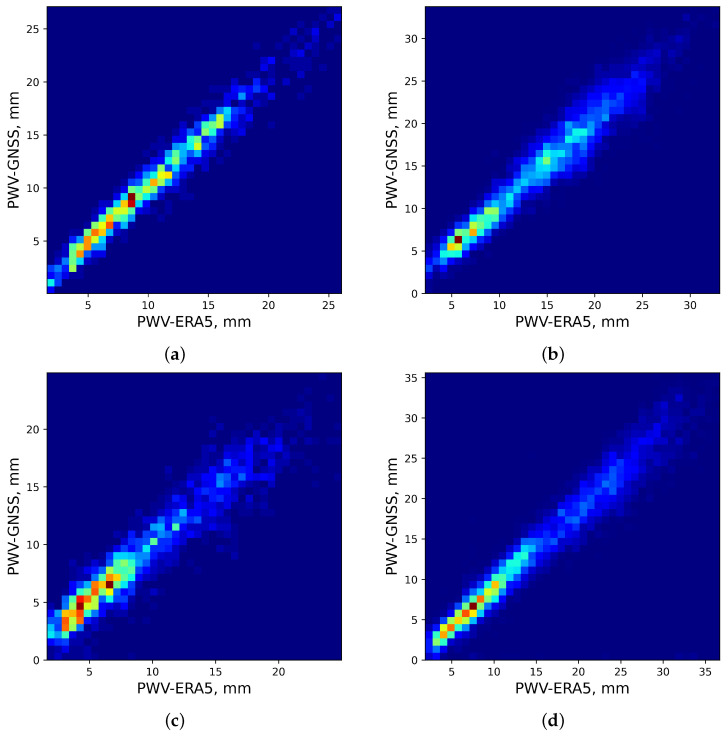
Two-dimensional distributions of PWV from GNSS and ERA-5. (**a**) Arti, height is 253.9 m a.s.l. (**b**) Aruch-Yerevan, height is 1199.2 m a.s.l. (**c**) Kars, height is 1756.0 m a.s.l. (**d**) Zelenchukskaya, height is 1143.4 m a.s.l. The red color corresponds to areas where values of PWV are most densely grouped.

In order to achieve accurate estimates of PWV, we correct the ERA-5 data using GNSS measurements. Examples of two-dimensional distributions of the ERA-5 reanalysis and GNSS values are shown in Figure 1. Table 1 provides the coordinates of GNSS stations. Analysis of the figure shows that the PWV values from the reanalysis and GNSS change synchronously. Especially, the values are closely related for the PWV < 10 mm. In this case, the Pearson coefficients are greater than 0.9. The areas with the highest point density correspond to the most typically observed atmospheric conditions for a given location. The dependencies obtained provide background information for use of the reanalysis data to assess astroclimate, particularly in calculation of precipitable water vapor statistics.

## 3. Atmospheric Characteristics Relevant for Millimeter Telescopes

### 3.1. Spatial Distributions of PWV Relevant for Placement of Millimeter Telescopes

Until now, the issue of site selection for new ground-based telescopes has not been resolved. At least, it has not been fully resolved for all sites on Earth with high astroclimatic characteristics. Moreover, global climate change may contribute to the degradation of conditions for existing astronomical observatories. First of all, this degradation is related to the increase in atmospheric water vapor content in recent decades.

Despite the fact that a number of sites with high astroclimatic characteristics are known in the world, identification of new locations and assessing local atmospheric characteristics are key tasks. The identification of new sites is very important for the expansion of the ground-based network of astronomical instruments: for example, at the moment, there are practically no ground-based telescopes in the Russian Federation that can perform observations at frequencies above 100 GHz [9]. Moreover, changing atmospheric conditions at some existing astronomical sites associated with global warming also make the solution of this problem relevant. In this study, we used ERA-5 reanalysis data for estimation of spatial distributions of precipitable water vapor. We found that the reanalysis data overestimate the PWV values, especially for mountain peaks. The ERA-5 data should be corrected. Correction of data based on consistency with measurements is a common procedure [32]. To obtain more accurate estimations of PWV, we use an empirical method based on taking into account the characteristic vertical changes in the water vapor content, calculated from data from nearby radiosonde or GNSS stations. In particular, the PWV values for a given grid node are corrected taking into account the exponential function:

(1) $$PWV=PWV_{0}exp\left(-0.439\Delta z/<H_{eff}>\right),$$

where $PWV_{0}$ is the value of PWV derived from ERA-5 reanalysis, and $<H_{eff}>$ is the average vertical scale of water vapor. $<H_{eff}>$ is such height of the lower atmospheric layer within which the water vapor content decreases with height above surface proportionally to the factor 1/exp. The value of Δz for a selected grid node is defined as the mean difference in heights within the local area around this reference node. In particular, to estimate Δz, we used the following expression:

(2) $$\Delta z=\frac{1}{M}\sum_{i=1}^{M}z_{n}\left(0\right)-z_{i}\left(\Delta x,\Delta y\right),$$

where $z_{nod}\left(0\right)$ is the height of the reference node, $z_{i}\left(\Delta x,\Delta y\right)$ are the heights of adjacent nodes shifted in horizontal directions by a distance Δx,Δy, and *M* is the number of nodes around the reference node. The shifts in Δx and Δy are typically about 100–120 km. The values are specified by minimizing the dispersion between the predicted and measured PWV values. Also, it should be noted that the PWV values have been corrected only for nodes with heights higher than 2000 m above sea level.

**Figure 2:**
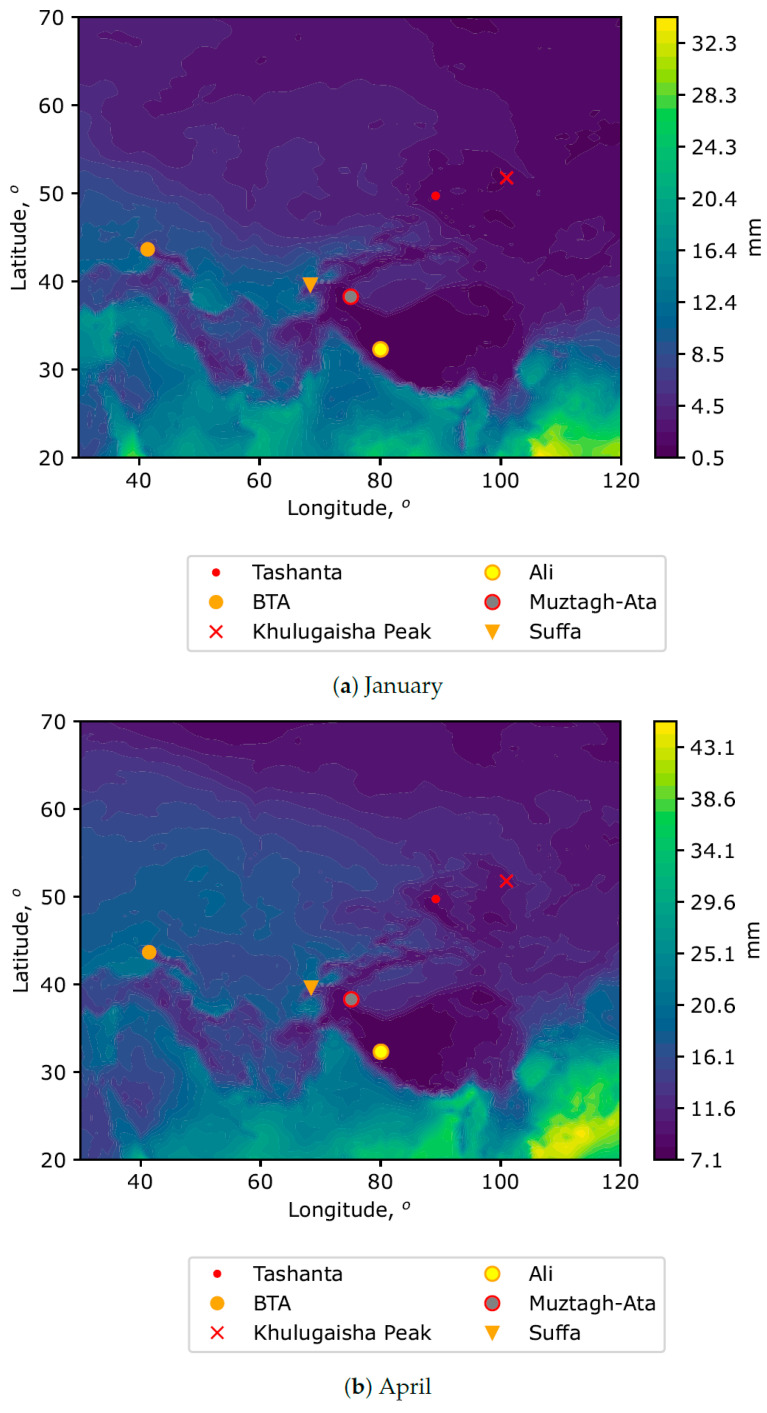

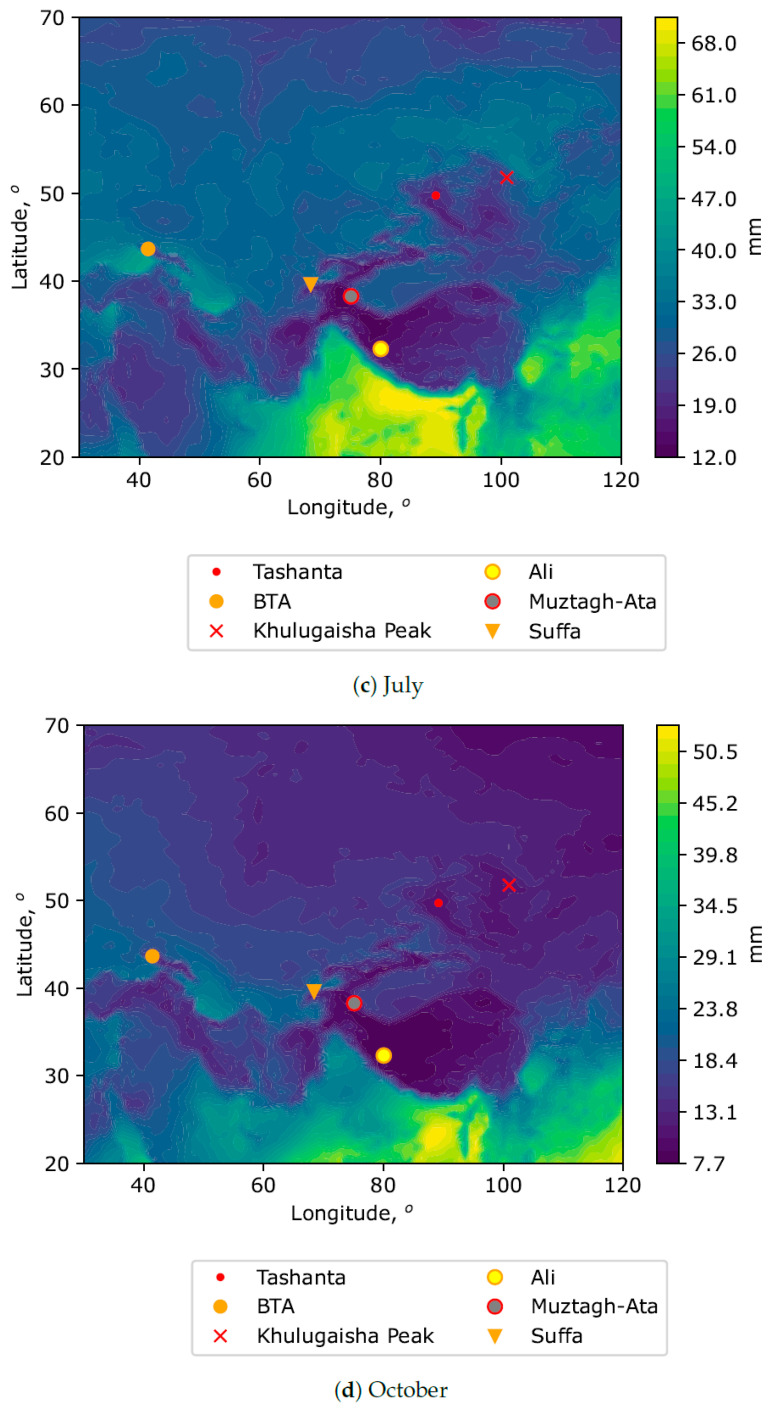
Spatial distributions of PWV medians for different months.

Figure 2 demonstrates the spatial distributions of median PWV averaged for the central months of seasons (January, April, July, and October).

Analysis of these figures shows that the precipitable water vapor is distributed unevenly within the selected macroregion. Low PWV values are observed in mountainous regions. The most interesting region is Tibet. In this area, a deep minimum of PWV is observed. Median values of PWV are below 2 mm. This corresponds to the most favorable conditions for millimeter observations. A certain configuration of zones is preserved throughout the year. It can be noted that the well-known astronomical sites (Ali-1, MuztaghAta, and Suffa) are located within these zones with low PWV. New sites (Khulugaisha Peak and Tashanta) are in stable locations throughout the year in terms of low PWV values.

**Figure 3:**
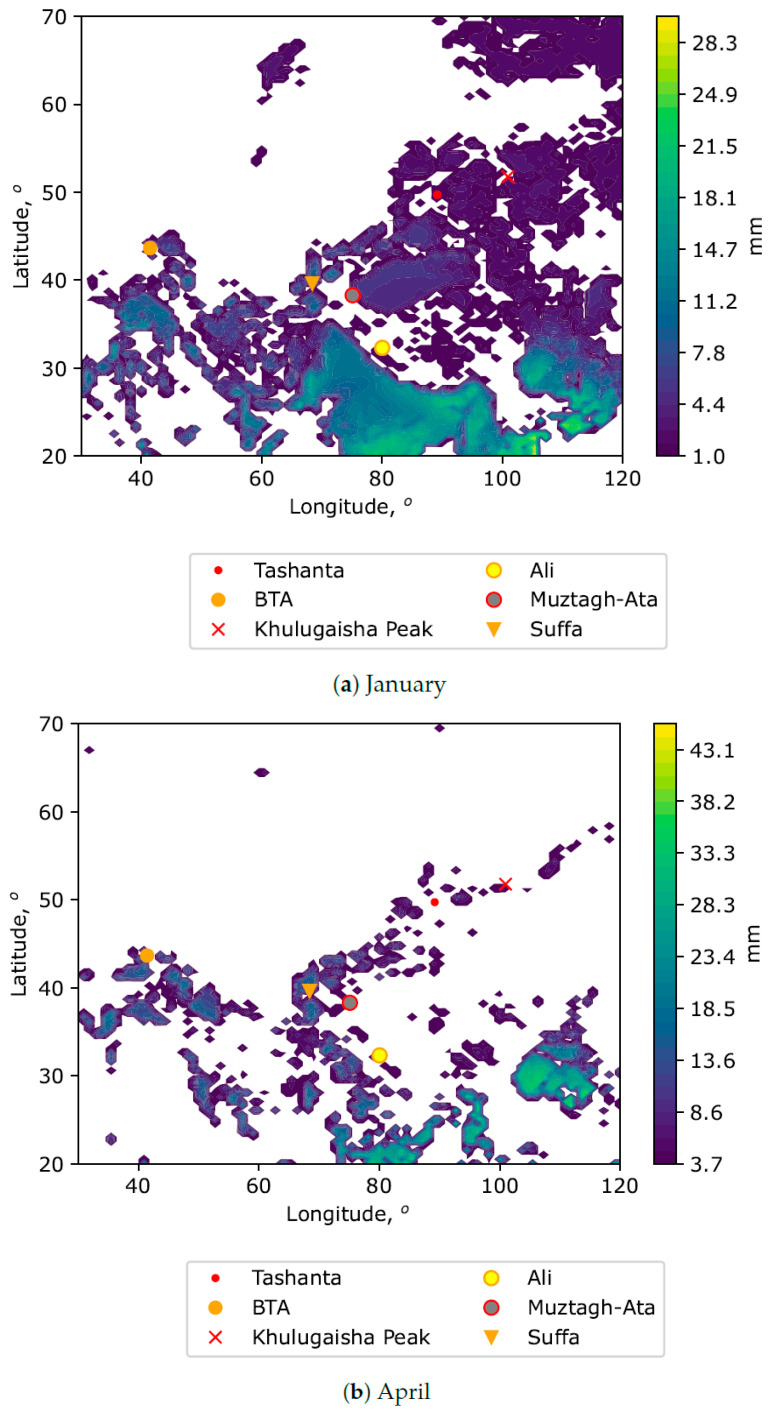

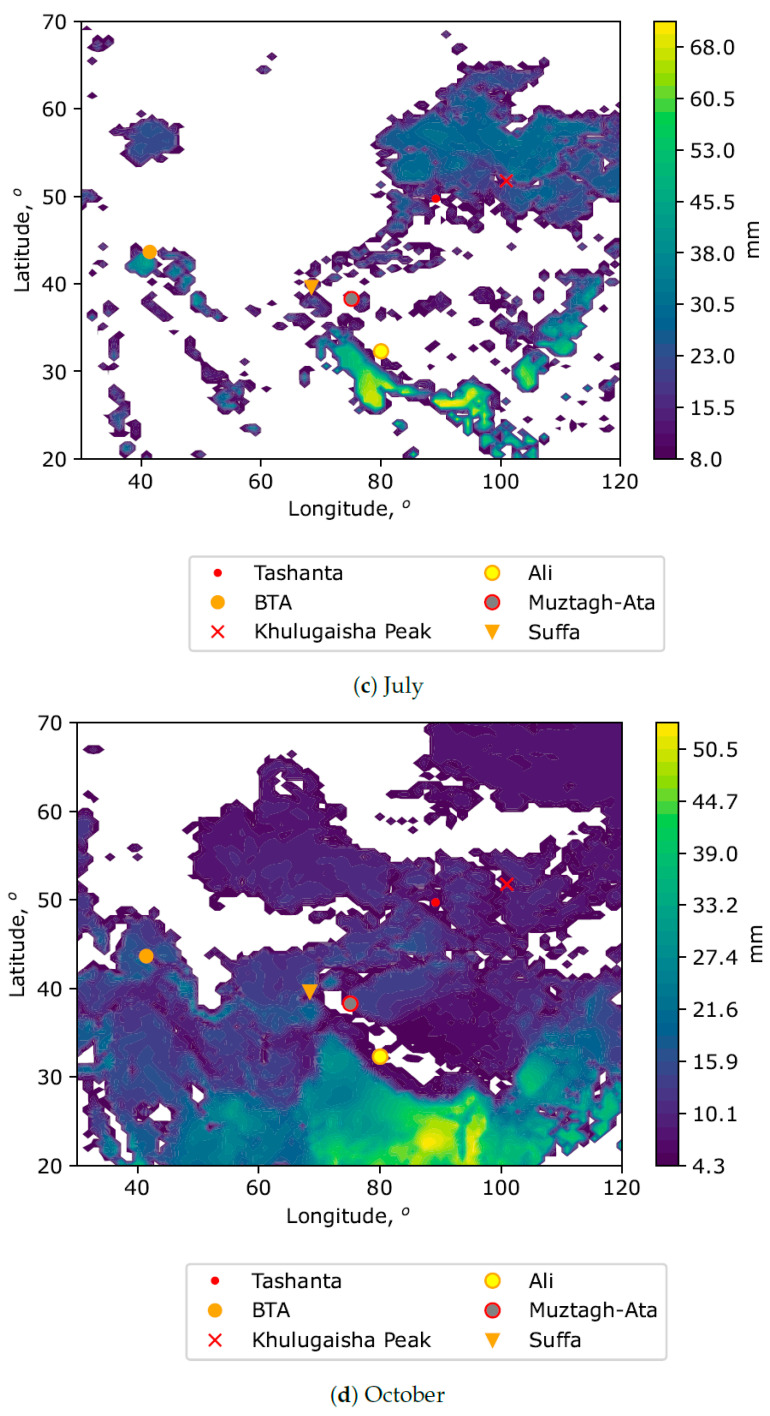
Spatial distributions of PWV medians for different months. Areas with strong wind gusts within the surface layer are excluded.

In addition to PWV, the possibility of placing a new submillimeter/millimeter telescope is also determined by the surface wind field. In this regard, we also provide averaged spatial distributions of median PWV values taking into account the surface wind speed (Figure 3).

In particular, areas with high values of wind gusts in the atmospheric surface layer (at a height of 10 m above the underlying surface) are excluded from the distributions. These figures make it possible to specify the optimal sites, both in terms of low PWV values and surface winds, leading to mechanical impact on the telescope structure. Analyzing the figures, it can be noted that all the presented astronomical sites (including Khulugaisha Peak and Tashanta) are located within the zones characterized by low PWV values and the absence of significant amplitudes of surface wind gusts.

Analysis of PWV at the sites of Khulugaisha Peak and Tashanta shows that the seasonal variations are similar. The minimum PWV is observed in the cold period of the year, and the maximum PWV corresponds to the warm period. Astronomical observations will be most effective in the period from October to April. The median PWV values in the cold period are 1.6 and 2.0 mm for Khulugaisha Peak and Tashanta, respectively. For the SAO, the most favorable period corresponds to medians of 4–4.7 mm. In order to compare these results, we also estimated PWV for some of the best sites, including Ali and Muztagh-Ata. Table 2 shows mean values of PWV at the best sites [17,33,34,35,36].

At the Ali site, analysis of radiosonde data provides a slightly lower median value of 1.08 mm [21]. Feng et al. [37] showed that 5% of the PWV values at the Ali location are less than 0.96 mm; the median is 1.63 mm. Our calculations show that the daytime median value based on Era-5 reanalysis data from September to April is 1.40 mm. At night, the median remains virtually unchanged for the same period and is 1.41 mm. At Muztagh-Ata, the PWV values determined for clear sky conditions by LHATPRO measurements in January–February 2018 were 0.52 mm [37]. The calculated annual average median PWV value based on Era-5 data is 1.98 mm and decreases to 0.79 mm in January–February.

The best conditions at Muztagh-ata site with daily median PWV less than 2 mm correspond to the period from October to March [38]. We will also provide some data for Suffa Observatory located on the periphery of the region with minimum values of PWV. Here, seasonal changes in PWV values are smoother. The PWV peak is in July–August and reaches 9.5 mm. Minimum values of PWV are also observed in winter. In January, the median PWV decreases to 2.5–2.7 mm.

The Muztagh-Ata site is an excellent high-altitude ground-based astronomical observing site [18]. Using the MERRA-2 data, the authors have estimated the water vapor content above the Muztagh-Ata site. The mean PWV values are 2.97, 5.99, 2.53, and 1.21 mm for spring, summer, autumn, and winter, respectively. Also, it is shown that the higher site in Ali (6100 m.a.s.l.) is on par with sites located at the Atacama Desert in northern Chile (∼5600 m.a.s.l.) in terms of transmission and stability. [39]. At the Suffa International Observatory, medians in PWV are higher compared to Muztag-Ata and Ali. The annual median PWV derived from Era-5 reanalysis is 5.4 mm. The best conditions are observed in December–January when the median values are close to 2.6–2.9 mm. The worst conditions are in July with a median of 8.9 mm.

### 3.2. Vertical Integrals of Water Vapor Fluxes

**Figure 4:**
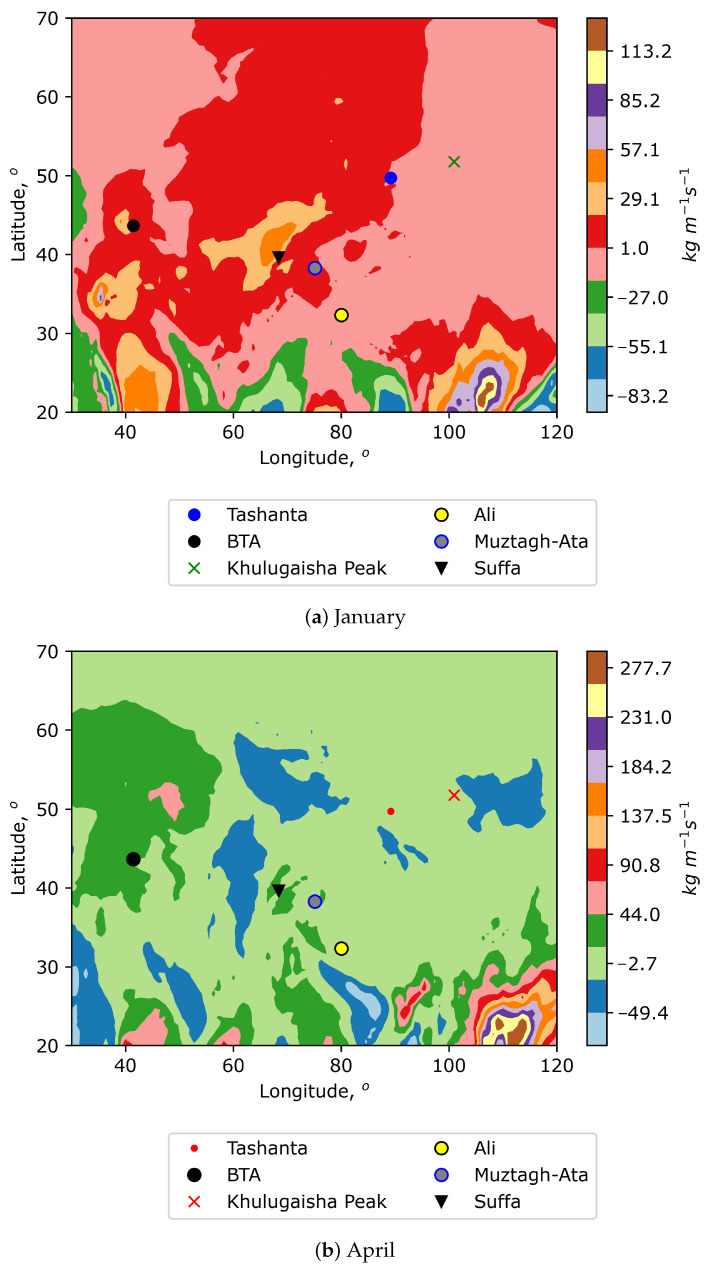

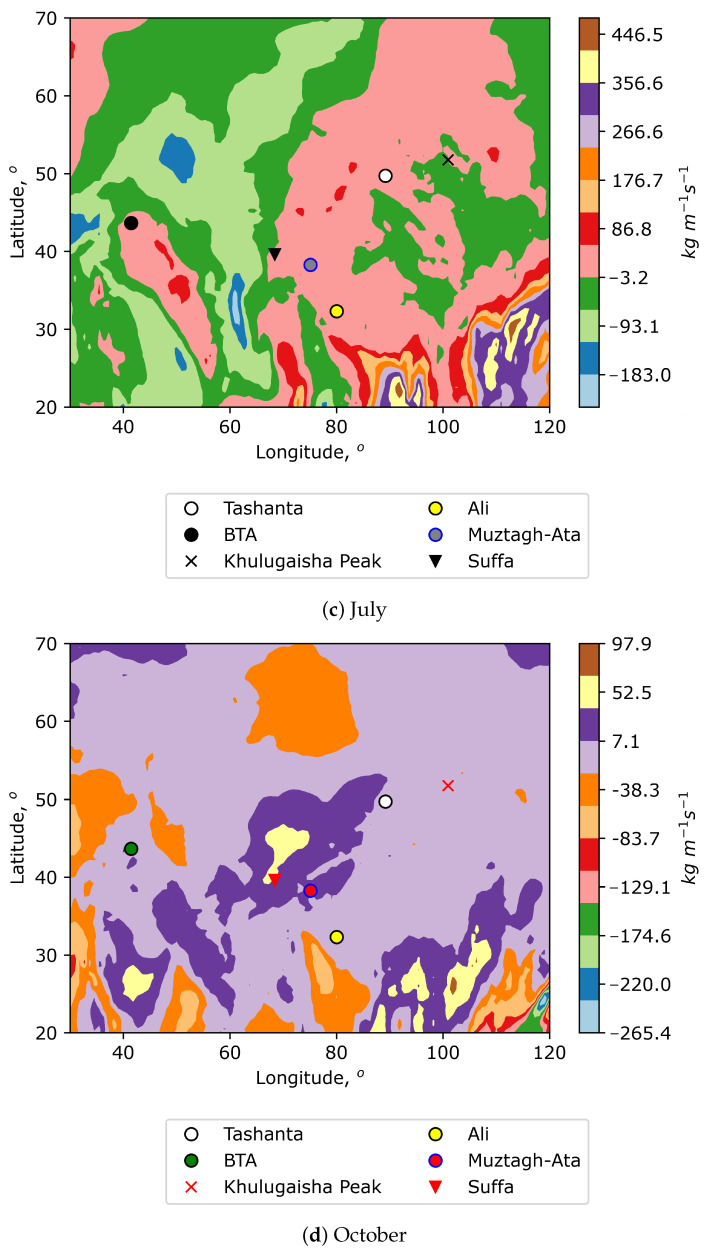
Spatial distributions of vertical integral of northward water vapor flux for different months. Positive values indicate a flux from south to north.

**Figure 5:**
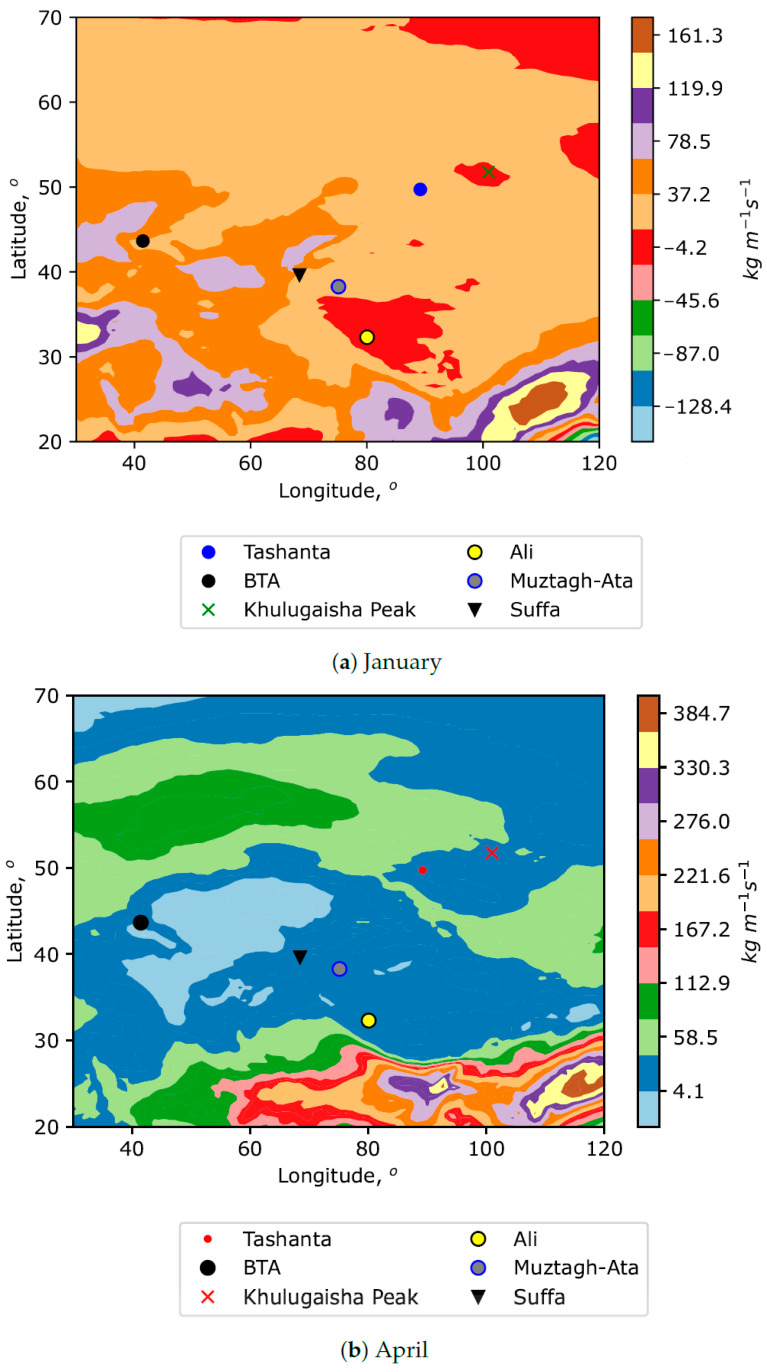

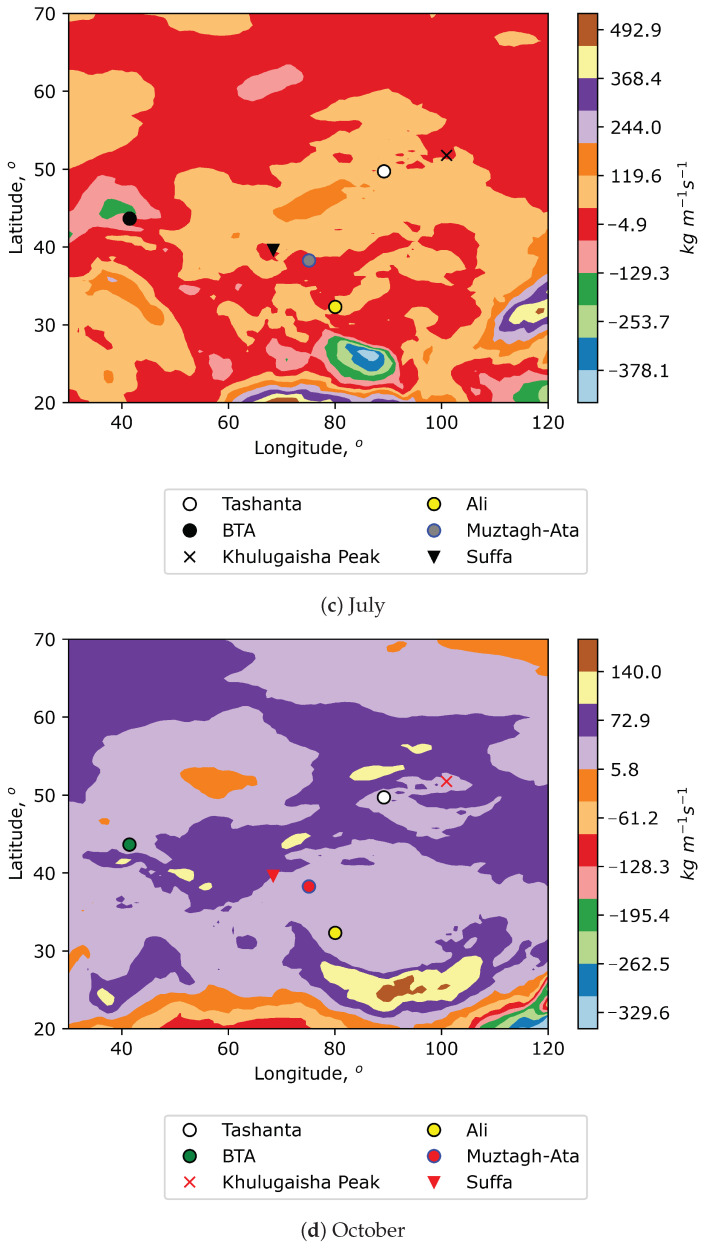
Spatial distributions of vertical integral of eastward water vapor flux for different months. Positive values indicate a flux from west to east.

Another interesting characteristic of atmospheric water vapor content is the vertical integral of water vapor flux. This quantity is a certain derivative function of PWV. Figure 4 and Figure 5 show eastward and northward water vapor fluxes for different months.

It characterizes the horizontal rate of flow of water vapor per meter across the flow for a column of air extending from the surface of Earth to the top of the atmosphere. Analysis of the figures shows that the selected locations are in areas with low and moderate (positive or negative values) of water vapor fluxes. Specifically, Khulugaisha Peak corresponds to areas of negative values of northward water vapor flux associated with colder and drier air masses. Tashanta is also located in areas of negative values of northward water vapor flux but of lesser intensity. Despite the fact that the figures show medians of fluxes, large amplitudes of values (with a certain degree of probability) characterize the power of the cold air flow (the source is often located remotely). Considering the north–south orientation of the air flow, a definite exception among the sites under consideration is the Suffa Observatory (in January). At this time, the southern air transfer prevails over the observatory. As for the power of the west–east air transfer, the figures show that the selected sites are in variable conditions. However, the locations are not subject to strong west–east air transfer. This indicates a greater contribution of local factors to astroclimatic conditions.

## 4. Machine Learning to Obtain More Accurate Values of PWV for Selected Astronomical Sites

Machine learning is a very useful tool in many scientific fields. Use of machine learning has demonstrated high efficiency in estimating PWV. For example, Khabarova et al. [40] suggested a method for determing precipitable water vapor from radiometric data using machine learning. Also, a non-meteorological model based on a neural network technique was developed for estimating PWV in the Austrian region [41].

In order to obtain more accurate estimates of precipitatable water vapor for the selected sites (Khulugaisha Peak and Tashanta), we propose to use the following approach. Considering the GNSS measurement data as aa reference, in the first step, we form an array of measurement data for a set of stations in the selected region. In our case, we selected Arti, Aruch-Yerevan, Kars, Kazan, and Zelenchukskaya. For prediction of PWV, we have used the input variables from reanalysis ERA-5: vertical profiles of air temperature and humidity, dew point temperature at Earth’s surface, and the height of the atmospheric boundary layer. Thus, the inputs of the neural network were meteorological characteristics from the reanalysis data, and the output was the measured value of PWV.

Using the Python 3.10.11 as well as TensorFlow 2.18 and Keras 3.0 packages, we create a number of neural networks that predict variations in PWV. PWV values, the heights of the atmospheric boundary layer, and vertical profiles of air humidity, extracted from the ERA-5 dataset, were selected as input parameters for training the neural networks. The key hyperparameters of the networks include the number of hidden layers, the number of neurons in each layer, and the activation function. The structure of each neural network was set before training, using a different number of layers, from 4 to 30. At the same time, the neural network model that provides the best agreement between measured and predicted values of PWV contains 10 layers, and the number of training epochs is 200. The number of neurons in each layer was variable: from 4096 in the first hidden layer to 32 in the last hidden layer. With an increase in the number of layers, the ability of the model to reproduce variations decreased despite an increase in the number of training epochs. For creation of the optimal network, we used linear activation functions. In our case, there was no need to use other activation functions since the approach used made it possible to refine the values of PWV.

**Figure 6:**
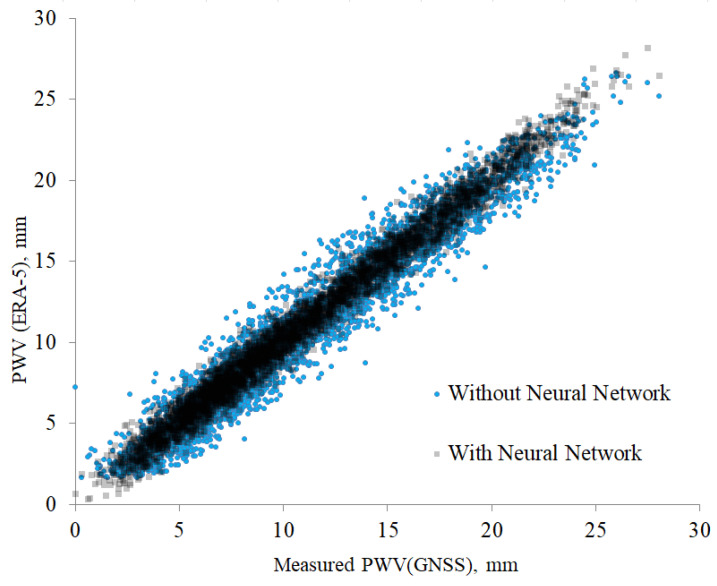
Deviations between measured and predicted values of PWV (neural network).

Figure 6 shows deviations between measured and predicted values of PWV. Analysis of the figure shows that the values of precipitable water vapor obtained with the neural network better correspond to the measured PWV. The root mean square (RMS) deviation of PWV values without using neural networks is 4.5 mm. With a neural network, the RMS is reduced to 2.8 mm.

## 5. Atmospheric Opacities with Millimeter-Wave Propagation Models

One of the key atmospheric characteristics for ground-based radio astronomy observations at short millimeter and submillimeter waves is opacity, primarily related to the water vapor and oxygen content in the atmosphere. Due to the limited measurements both in time and space, atmospheric models play a major role in estimation of atmospheric opacity. In particular, we used well-known models (LIEBE, HITRAN/PARDO, and JPL/PARDO) for calculation of atmospheric opacity above new sites (Tashanta and Khulugaisha) and the Special Astrophysical Observatory (SAO). The calculated values of opacity depend on the spectral line (line-by-line model) characteristics and an empirical continuum due to water vapor and dry air. In the study, we provided three opacities:

(1) JPL/Pardo-derived values. The calculations are based on a continuum absorption derived by Pardo J. from transmission observations over Hawaii astronomical observatory [42]. The line-by-line model uses lines described in the JPL catalogue (2008) and completed with HITRAN (version 2008).

(2) HITRAN/Pardo model. The model uses the same Pardo continuum but with spectral lines described in the HITRAN-2008 catalogue.

(3) Liebe model. The model is based on lines and continua generated and presented by Liebe at al. [43].

**Figure 7:**
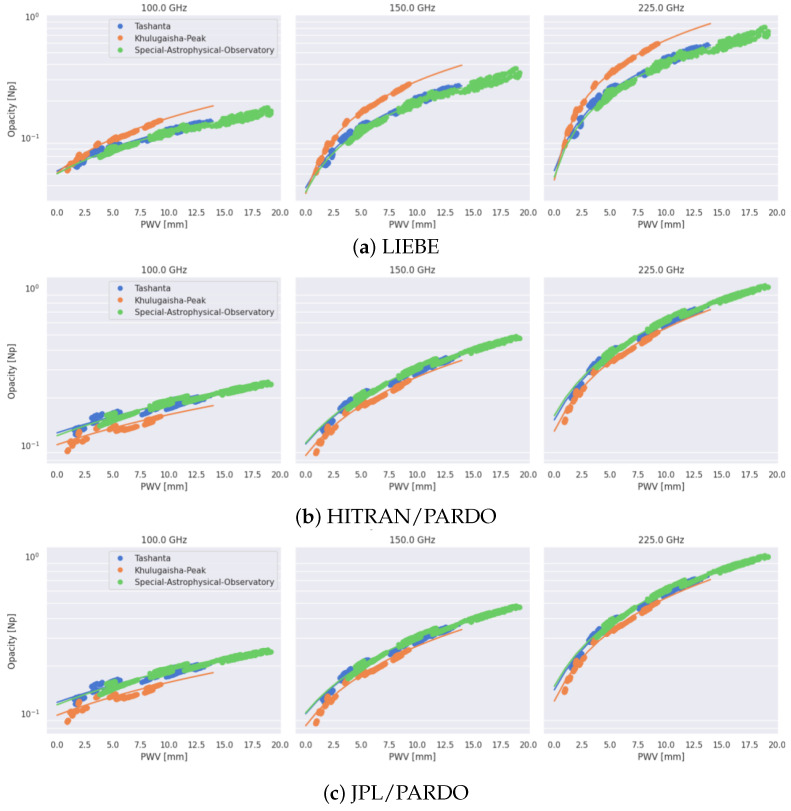
Atmospheric opacities at the sites suitable for millimeter observations.

Figure 7 shows the dependencies of atmospheric opacity on precipitable water vapor for three sites with high astroclimatic characteristics. For the best sites within Russia, seasonal changes in atmospheric opacities are shown in Table 3. Analyzing the presented data, we can conclude that Khulugaisha Peak has better conditions for millimeter (and also submillimeter) observations in comparison with Tashanta. Also, we can consider that Pardo-based models are better in the submillimeter domain and that Liebe is better below 150 GHz.

## 6. Conclusions

Plans to develop a global network of millimeter/submillimeter telescopes generate the need for detailed comprehensive studies of the astroclimate and the identification of new astronomical sites. The Eurasian Submillimeter Telescopes (ESMT) project concept envisages the deployment of three new mm/submm 15–21 m telescopes. The selection of a site for a new millimeter/submillimeter telescope is always a complex, multi-faceted task. It is largely determined by the atmospheric properties, specifics of the designed telescope, and its technical equipment. At present, there are no calibrated data on the spatial distribution of precipitable water vapor and on the optical opacity of the atmosphere within the northern part of Eurasia. The practical aspect of this study is that new information relevant to the selection of a specific site for a telescope has been obtained.

The following is a conclusive summary. This paper focuses on the determination of the distributions of PWV within a selected macroregion and the estimation of the optical opacity of the atmosphere for selected sites for the deployment of new submillimeter/millimeter telescopes. In particular, the statistical analysis of PWV in the locations of interest allowed us to obtain the following conclusions:

(i) Averaged spatial distributions of PWV for January, April, July, and October are determined. Areas with high wind gust values within the surface layer of the atmosphere are excluded from these distributions. In perspective, these distributions will make it possible to identify new sites for ground-based millimeter/submillimeter telescopes.

(ii) In order to improve the accuracy of PWV estimations, it is proposed to use deep neural networks based on the ERA-5 reanalysis data. It is shown that the deviation decreases by 1.6 times.

(iii) High absolute altitudes combined with low air temperatures in the locations of Khulugaisha Peak and Tashanta result in low water vapor content in the atmospheric column. This is especially pronounced during the cold period. The sites are located within areas with high astroclimatic characteristics. The dependencies of the optical opacity on PWV for Khulugaisha Peak, Tashanta, and the Special Astrophysical Observatory are obtained. These dependencies can be used to estimate the average values of the optical opacity for different frequencies (100 GHz, 150 GHz, and 225 GHz). The diagrams also make it possible to determine the inversion points where the opacity values estimated for different frequencies become equal to each other.

(iv) Seasonal changes in absorption coefficients for water vapor and oxygen are estimated. These estimates, obtained from the modeling results, can be useful in organizing and planning field measurements in certain time intervals. We can conclude that Khulugaisha Peak has better conditions for placing a millimeter telescope in comparison with Tashanta. The latter makes it possible to recommend Khulugaisha Peak for the placement of the submillimeter telescope. It is for this site that we expect high repeatability of time intervals with a consistently low content of water vapor in the atmospheric column (and along the line of sight).

(v) It is important to emphasize that the Liebe model better describes the transparency of the atmosphere for 100 GHz. For frequencies of 225 GHz and higher, preference can be given to the JPL/Pardo and HITRAN/Pardo models.

## Figures and Tables

**Table 1:** Coordinates of GNSS stations.

Station	Coordinates
Arti	$58^{\circ }33^{\prime }37^{\prime \prime }$ N$,\;56^{\circ }33^{\prime }47^{\prime \prime }$ E
Aruch-Yerevan	$44^{\circ }5^{\prime }8^{\prime \prime }$ N$,\;40^{\circ }17^{\prime }9^{\prime \prime }$ E
Kars	$43^{\circ }5^{\prime }36^{\prime \prime }$ N$,\;40^{\circ }35^{\prime }18^{\prime \prime }$ E
Zelenchukskaya	$41^{\circ }33^{\prime }54^{\prime \prime }$ N$,\;43^{\circ }47^{\prime }18^{\prime \prime }$ E

**Table 2:** Mean values of PWV at the best sites.

Site	PWV, mm (January)	PWV, mm (July)	Data
Ali	0.8	4.8	ERA-5
Lenghu site	1.1	6.5	MERRA-2
Lenghu site	1.2	8.6	ESO website
Mauna Kea	1.9	3.9	ESO website
Cerro Paranal	2.1	4.3	ESO website
La Palma	3.8	6.1	ESO website
Tashanta	1.9	12.5	ERA-5
Khulugaisha Peak	1.6	9.8	ERA-5
Muztagh-Ata	1.2	6.8	ECMWF data
Muztagh-Ata	1.2	6.8	ECMWF data
Suffa	2.6	9.5	ERA-5
Maidanak	3.2	9.1	GPS
Sanglokh	3.0	9.8	ERA-5
Koluch-Kul	1.4	7.8	ERA-5

**Table 3:** Atmospheric opacities at the sites suitable for millimeter observations: Khulugaisha Peak and Tashanta.

		Khulugaisha Peak			Tashanta	
	**100 GHz**	**150 GHz**	**225 GHz**	**100 GHz**	**150 GHz**	**225 GHz**
**Month**	**Opacity**	**Opacity**	**Opacity**	**Opacity**	**Opacity**	**Opacity**
LIEBE						
1	0.06	0.07	0.12	0.07	0.08	0.14
2	0.06	0.06	0.11	0.06	0.06	0.10
3	0.07	0.09	0.17	0.08	0.10	0.18
4	0.09	0.14	0.28	0.09	0.12	0.25
5	0.10	0.16	0.34	0.12	0.21	0.46
6	0.13	0.24	0.52	0.12	0.22	0.48
7	0.12	0.24	0.51	0.12	0.22	0.48
8	0.10	0.19	0.40	0.11	0.20	0.42
9	0.10	0.17	0.35	0.10	0.16	0.33
10	0.07	0.10	0.19	0.08	0.11	0.23
11	0.07	0.08	0.15	0.07	0.09	0.18
12	0.05	0.05	0.09	0.06	0.06	0.11
HITRAN						
1	0.10	0.10	0.17	0.13	0.14	0.23
2	0.11	0.10	0.17	0.13	0.13	0.20
3	0.12	0.13	0.22	0.15	0.17	0.30
4	0.13	0.17	0.30	0.16	0.21	0.38
5	0.13	0.18	0.35	0.19	0.32	0.64
6	0.14	0.23	0.47	0.20	0.34	0.69
7	0.14	0.23	0.45	0.19	0.34	0.70
8	0.13	0.19	0.37	0.18	0.31	0.63
9	0.12	0.18	0.35	0.17	0.26	0.51
10	0.11	0.13	0.23	0.15	0.19	0.36
11	0.11	0.11	0.19	0.14	0.17	0.29
12	0.09	0.09	0.14	0.12	0.12	0.20
JPL						
1	0.10	0.10	0.17	0.12	0.14	0.23
2	0.10	0.10	0.16	0.12	0.12	0.20
3	0.12	0.13	0.21	0.15	0.17	0.29
4	0.13	0.16	0.29	0.16	0.20	0.37
5	0.13	0.18	0.34	0.20	0.32	0.63
6	0.14	0.23	0.46	0.20	0.34	0.68
7	0.14	0.22	0.44	0.20	0.34	0.69
8	0.12	0.19	0.36	0.18	0.31	0.62
9	0.12	0.18	0.34	0.17	0.26	0.50
10	0.11	0.13	0.22	0.15	0.19	0.35
11	0.10	0.11	0.19	0.14	0.16	0.29
12	0.09	0.09	0.14	0.11	0.12	0.19

## Data Availability

Data used are available on request from the corresponding author.
